# Serum-Free Medium for Recombinant Protein Expression in Chinese Hamster Ovary Cells

**DOI:** 10.3389/fbioe.2021.646363

**Published:** 2021-03-15

**Authors:** Weifeng Li, Zhenlin Fan, Yan Lin, Tian-Yun Wang

**Affiliations:** ^1^Department of Biochemistry and Molecular Biology, Xinxiang Medical University, Xinxiang, China; ^2^International Joint Research Laboratory for Recombinant Pharmaceutical Protein Expression System of Henan, Xinxiang Medical University, Xinxiang, China

**Keywords:** cell culture, recombinant therapeutic protein, Chinese hamster ovary cells, serum free medium, glycosylation

## Abstract

At present, nearly 70% of recombinant therapeutic proteins (RTPs) are produced by Chinese hamster ovary (CHO) cells, and serum-free medium (SFM) is necessary for their culture to produce RTPs. In this review, the history and key components of SFM are first summarized, and its preparation and experimental design are described. Some small molecule compound additives can improve the yield and quality of RTP. The function and possible mechanisms of these additives are also reviewed here. Finally, the future perspectives of SFM use with CHO cells for RTP production are discussed.

## Introduction

Chinese hamster ovary (CHO) cells are the preferred expression system for recombinant therapeutic protein (RTP) production, and nearly 70% of RTPs are currently generated by CHO cells (Pereira et al., 2018; Stolfa et al., 2018). In particular, CHO cells are used to produce recombinant therapeutic proteins and monoclonal antibodies (mAbs), such as adalimumab, bezlotoxumab, avelumab, dupilumab, durvalumab, ocrelizumab, and brodalumab (Kaneyoshi et al., 2019a; Henry et al., 2020). CHO cells have the following advantages over other systems: (1) they are not susceptible to human virus infection and have high safety (Lalonde and Durocher, 2017); (2) post-translational modifications are similar to those in human cells. Glycosylation of CHO glycoprotein is closer to that in human cells due to the lack of immune galactose-galactose epitopes in CHO cells (Stach et al., 2019); (3) exogenous proteins are easily synthesized and secreted into the medium; (4) cultured in adhesive or suspended state; and (5) the cell density can be higher under serum-free culture (Ritacco et al., 2018; Dahodwala and Lee, 2019). The common CHO cell culture usually requires 10% fetal bovine serum, which can increase the risk of contamination and be unfavorable for protein purification during the downstream process (Kim et al., 2020). Hence, serum-free medium (SFM) for CHO culture is necessary for RTP production.

## Type and Key Components of Serum-Free Media

### Types of Serum-Free Media

The culture media used in the earliest animal-derived cell cultures were composed of serum (human or fetal bovine), plasma, or tissue extracts of nutrient-like components, which were complex, indefinite composition, and prone to increase the risk of contamination (Lalonde and Durocher, 2017). CHO cell culture medium underwent three stages of development: natural medium, synthetic medium, and SFM (Glassy et al., 1988). There are five types of SFM that maintain cell growth without animal serum: common SFM, Xeno-free medium, animal-free medium, protein-free medium, and chemically defined medium (Yao and Asayama, 2017; Gélinas et al., 2019). The development and main components of SFM are shown in Table 1.

**TABLE 1:** Development and characteristic of SFM.

**Type**	**Characteristic**
SFM	A variety of biological materials that can replace the function of serum, contains a large number of plant and animal protein and unknown components, such as bovine serum albumin or α- or β-globulin, used as a supplement.
XFM	Human-source components, such as human serum albumin, are used as supplements but animal components are not allowed as supplements.
AFM	No animal source proteins, and the required proteins are derived from recombinant proteins or proteolytic proteins.
PFM	No serum at all, no protein or low protein content, contains undefined components such as peptide components (hydrolyzed proteins).
CDM	No serum, no protein, chemical defined component; suitable for different cells growth and recombinat protein production.

### Key Components of Serum-Free Media

In the last 20 years, with the widespread application of SFM in the large-scale culture of animal cells, SFM has become a research hotspot, and some progress has been made in the following three aspects: (1) more comprehensive and in-depth understanding of the nature and role of the key components commonly used in SFM (Rodrigues et al., 2012), (2) development of traditional SFM to protein-free medium (Devireddy et al., 2019), and (3) development of a chemically defined medium (Jenkins et al., 1994). To develop a chemically defined medium, first, it is necessary to determine its specific components as well as identify the specific nutrients and minimum content to support cell growth (Jenkins et al., 1994). The glycosylation profile of therapeutic mAbs is the critical quality attribute for industrial immunoglobulin G (IgG) production. Several alternative carbon sources, which function as glycosylation precursors, can influence glycosylation patterns. Since cells preferentially consume glucose, the presence of this substrate reduces the effect of substitution sugars on glycosylation.

### Energy Sources

Glucose is the most commonly added sugar in the cell culture process and is an indispensable substance as the main energy source of cells in the culture medium (Fan et al., 2015). During the cell culture process, high concentrations of glucose are usually consumed by the cells in a low-efficiency anaerobic respiration pattern, which can generate a considerable amount of lactic acid to inhibit cell growth. Fructose, galactose, and other sugars have been used to replace some of the glucose to improve its utilization rate during culture and to control the production of lactic acid during the cultivation process (Fan et al., 2015; Zhang et al., 2019). Mammalian cells can utilize oligosaccharides instead of glucose as a carbon source, which can reduce lactic acid production. The specific maltose consumption in CHO cells is similar to that of galactose and fructose. CHO cells can grow normally under higher maltose concentrations. Furthermore, the application of a maltose supplement to produce recombinant mAbs in batch and batch feed culture has been verified. The titers of recombinant mAbs were found to increase by 15 and 23%, respectively, and no obvious adverse reaction to the glycosylation spectrum of the antibody was identified (Leong et al., 2018). Combinations of mannose, fructose, galactose, and fucose were added to the medium when glucose was depleted and lactate accumulated in CHO cells. The results showed that mannose or glucose decreased high-mannose glycan levels by 3–7%, galactose increased the levels of monogalactoglycans (G1) by 8–20% and digalactoglycans (G2) by 2–6%, and fucose showed a significantly higher concentration of intracellular GDP-fucose (Zhang et al., 2019).

### Amino Acids

Amino acids are the most important sources of nitrogen in cell culture media, and some are also important energy sources, such as by being sources of glutamine and asparagine (Fan et al., 2015). According to the characteristics of mammalian cell metabolism, amino acids can be divided into essential and non-essential amino acids (Kato et al., 2018). Different cell lines have different requirements for amino acids. Reasonably optimizing the ratio of various amino acid concentrations is an effective way to increase the density of living cells and the expression level of recombinant proteins during the culture process (Zhang et al., 2015). Addition of non-essential amino acids to the medium has a positive effect on the production of biomass, in which cysteine and tyrosine play a fundamental role. The extracellular availability of non-essential amino acids limits the extent of energy loss in the amino acid biosynthesis pathway and provides additional reductive power to other biological processes (Yao and Asayama, 2017).

### Vitamins

Vitamins are key components in SFM and important for maintaining cell growth and play a regulatory and controlling role in cell metabolism. They can be classified as water- and fat-soluble vitamins (Burer et al., 2020). The demand for vitamins in different cell lines varies widely and needs to be targeted for optimization. The addition of vitamins to CHO cell media can increase the yield of mAbs by up to three times, but not all vitamins are important for cell growth (Bai et al., 2021). Many vitamins are easily decomposed by air oxidation, heat, and light, such as the commonly used ascorbic acid and tocopherol, which are sensitive to air oxidation, and ascorbic acid, thiamine, riboflavin, and cobalamin, which are sensitive to light. Therefore, protection from light and low temperatures is essential during the storage of the medium.

### Lipids

Lipids can be used to store energy in cells and are important constituents of the cell membrane structure (Budge et al., 2020). In addition, lipids are involved in the cell signaling processes. Among unsaturated fatty acids, linoleic and linolenic acids cannot be synthesized by cells and must be taken up from the culture medium. Ethanolamine and choline can promote cell proliferation. Some studies have found that lipids can effectively increase protein expression and glycosylation (Jenkins et al., 1994; Dean and Reddy, 2013; Budge et al., 2020). The glycosylation level of interferon expressed by CHO cells was effectively increased by adding the lipid mixture to the medium (Jenkins et al., 1994).

### Trace Elements

Trace elements are essential nutrients for cell metabolism and are the main additions to SFM. Moreover, they are involved in the enzymatic reaction of cells, which can regulate the activity of enzymes (Betts and Dickson, 2017). Copper (Cu), iron (Fe), manganese (Mn), molybdenum, nickel (Ni), selenium (Se), silicon (Si), zinc (Zn), and others are commonly used trace elements in culture medium. Among them, Fe, Se, Cu, and Zn have significant effects. Fe is an essential element related to many enzymes involved in DNA replication and cell metabolism. Fe deficiency can cause the cell cycle to block in G0 or G1 or cause fast-dividing cells to enter apoptosis (Xu et al., 2018). Ferric citrate is generally considered a substitute for ferritin, but a low concentration of ferric citrate is favorable for CHO cells to adhere to the micro-carrier, while a high concentration of ferric citrate is favorable for CHO cells to grow in suspension (Xu et al., 2018). Se is an important component of proteins that can improve the antioxidant capacity of cells. It is also a cofactor for glutathione peroxidase, which protects cells from damage by reactive oxygen species (Zhang et al., 2006). Zn can replace growth factor (GF) and stimulate cell growth. When 60 μM of ZnSO_4_⋅7H_2_O was added to medium, the yield of mAb increased by 2.0 and 6.5 times, respectively (Kim and Park, 2016).

### Anti-Shear Protectant

Cells are subjected to shear forces during suspension culture. Serum can reduce the shearing force on cells and protect them (Elias et al., 1995). In SFM, plutonic F-68 surfactant is commonly used to prevent cell damage at 0.3–2 g/L concentration (Zhang et al., 1992).

### Inorganic Saltion

Inorganic salt components in the medium mainly include sodium, potassium, magnesium, calcium, and phosphorus. Their main function is to help maintain a stable osmotic pressure during the cell culture process because each cell line has its tolerable osmotic pressure range (Qin et al., 2019). In addition, inorganic salt ions are involved in many cellular metabolic regulations. In CHO cells, inorganic phosphate limitation can cause another metabolic pathway with higher dependence on glucose availability, exhibiting higher growth and higher pyruvate carboxylase flux (Maralingannavar et al., 2017).

### Nucleic Acid Substances

Animal cells can generally synthesize nucleotides by *de novo* synthesis without the need to add exogenous nucleic acids to the culture medium. However, for cells whose *de novo* synthesis pathway is blocked, such as dihydrofolate reductase-deficient cells, it is necessary to add substrates for the remedial pathway, namely hypoxanthine and thymidine (Carvalhal et al., 2003). The addition of 1 mmol/L adenine (or 1 mmol/L guanine) can severely inhibit the growth of CHO cells, and 5 mmol/L uracil (or 10 mmol/L cytosine) does not affect the growth of CHO cells (Wong et al., 2010; Morrison et al., 2019). The different effects of nucleic acids on cell growth may be attributed to the different cell lines and culture processes used. In addition, the use of nucleic acids to increase antibody glycosylation has been reported. The addition of uracil can effectively increase the galactosization level of the antibody, which effectively increases the level of nucleoside sugar by combining the nucleic acid with the precursor substance (Yang et al., 2017; Kuwae et al., 2018).

### Other Ingredients

Furthermore, during the development of SFM, many functional additives were identified, including small molecule substances that can stimulate the expression of antibodies (Pegg, 2016). These substances are mainly divided into the following two categories: (1) carboxylic acids, of which sodium butyrate (NaB) is the most typical representative. Overall, 5 mmol/L NaB can increase the specific production rate of antibodies in low-yielding cell lines and increase the specific production rate of antibodies in high-yielding cell lines by nearly two-fold (Lim et al., 2019); and (2) antioxidants such as ascorbic acid and reduced glutathione. The expression rate of the tissue type plasminogen activator can be increased by adding antioxidants (ascorbic acid and reduced glutathione) to the CHO cell culture process (Allen et al., 2008). The main components of SFM are shown in Table 2.

**TABLE 2:** The composition of SFM.

**Category**	**Function**	**Example**	**Substitute**
Energy sources	Carbon sources	Glucose	Fucose/Galactose/Mannose/Maltose/Gluconeogenic pathway etc.
Amino acids	Nitrogen sources	EAA/NEAA (Asparagine/) Glutamine	Synthetic amino acid/Peptide compound.
Vitamin	Maintains cell growth	WSV/FSV (vitamin C/E)	
Lipid	Energy storage substance/signal transduction	SFA/USFA	Linoleic acid/Oleic acid/Ethanolamine/Choline etc.
Trace elements	Cell metabolism/Enzyme	Fe/Se/Cu/Zn	
Anti-shear protectant	Prevent cell damage.	Shearing force/P-F68	Surfactant/Reduce shear force.
Inorganic saltion	Stable osmotic pressure	Na/K/Mg/Ca/P	Cell physiological activities/Intercellular substance/Cell metabolism/Physiological functions.
Nucleic acid substances	Signaling pathway blocking	Hypoxanthine/Thymidine	
Others	Promoting cell growth and protein expression	Transferrin/Fe/growth factor.	NaB/antioxidants; Specific production/expression rate of tPA.

## Preparation and Experimental Design of Serum-Free Medium

**FIGURE 1:**
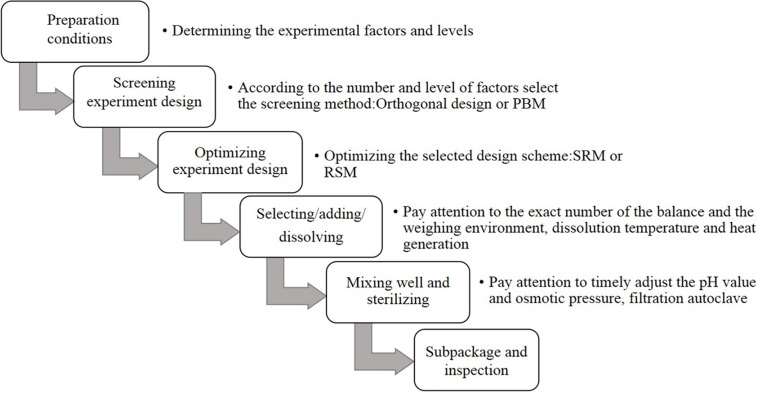
Optimization and preparation of serum-free culture screening process.

For the preparation process and optimization design of CHO SFM, first, the overall requirements of the raw materials and equipment are determined. The most basic components of CHO SFM include amino acids, inorganic salts, and vitamins, with little difference in cost. In addition, some special trace elements and lipid mixtures need to be further optimized, which requires high cost. Future studies should aim at reducing the overall cost by beginning with the optimization of these two categories of substances (Raab et al., 2010; Xiao et al., 2014; Kimiz-Gebologlu et al., 2020). Among them, the type and culture mode of the bioreactor also affect the quality of the final product considerably. In recent years, a large number of studies have investigated high-performance SFM and technology, which have promoted cell culture considerably. The methods used in the development of SFM mainly involve chemical analysis, statistical analysis, and molecular biotechnology for the culture process (Figure 1; Allen et al., 2008; Miki and Takagi, 2015; Hiller et al., 2017).

### Culture Process Analysis

The culture process analysis method is based on cell metabolism because various nutrients must be maintained at certain levels throughout. Through real-time monitoring of glucose, amino acids, and vitamins, fatty acids and trace elements in the cell culture process can appropriately adjust each medium component (Zhang et al., 2013). Based on real-time detection of certain nutrients in the cultivation process, nutrients can be classified into two groups, and specific consumption/generation rates can be calculated, rapidly depleting and non-depleting (Xu et al., 2014). Therefore, by continuously enriching the fast-consumptive nutrient concentration in the continuous culture process, a better steady-state culture effect can be achieved (Ghasemi et al., 2019). Monitoring cell growth is critical to the success of the animal cell culture process and can be accomplished by a variety of direct and indirect means. In most cases, glucose is the primary source of carbon and energy for cultured mammalian cells, but glycolytic metabolism often leads to lactic acid accumulation. Therefore, glucose and lactic acid levels are routinely measured to determine the metabolic activity of the culture. Although the metabolic rate of glucose or lactic acid varies with the culture stage, the cumulative consumption of the combination of glucose and lactic acid is linearly related to the living cell integral, and the slope indicates the specific consumption rate of the combination of glucose and lactic acid. Other studies have also shown that the cumulative consumption rate remains relatively stable under different culture conditions. The insensitivity of the cumulative consumption rate to process changes allows the simple and accurate determination of living cell density by measuring glucose and lactic acid (Tsao et al., 2005; Xing et al., 2009).

Quantitative PCR and protein electrophoresis are used to analyze changes in the mRNA and protein levels of each enzyme involved in the metabolism process in the cell, and some additional components are specially added to the culture medium to regulate the physiological metabolism of the cells (Torkashvand et al., 2015). Ganne and Mignot (1991) effectively controlled cell proliferation, apoptosis, and transgene expression by regulating the cell physiological metabolism through adding small molecules to the medium.

### Design of Experiment

Good design of experiment (DOE) can simplify experimental procedures and improve efficiency by the optimization of multiple components and their interactions, reducing the number of experiments and workload. The design ranges from a wide range of full factor design to several factor designs with at least two levels of evaluation. Among all possible combinations, partial factor design analyzes only one subset. More complex models, such as Plackett Burman method (PBM) and response surface method, can identify important and some interactions to reduce the total number of experiments. PBM is the most widely used in fractional factorial design. It is a two-level experimental design method that can perform the minimum number of experiments to quickly and reliably screen the main influencing factors from a large number of factors (Mohamad et al., 2020). PBM has been used to design the conduction of 24 groups of experiments, and results showed that 20 types of components can promote cell growth and increase the recombinant protein yield by 45% (Kato et al., 2018). The response surface method can achieve the best response effect by modeling and function fitting for multiple factors of interest and considering the interaction between each factor. However, it can only analyze a limited number of factors (generally <5).

Non-parametric Gaussian kernel regression and partial least squares regression were used to perform normalized fitting for data from 50 mixed media (Zou et al., 2020). Then, their selection accuracy was evaluated by analysis of variance. Next, the relationship between the key components identified by the non-parametric Gaussian kernel regression algorithm and mAb titers was studied through univariate effect analysis (Zou et al., 2020). The use of multiple statistical methods has become increasingly popular. Multiple design has become the preferred design method to optimize multi-factor level experiments, which is conducive to the optimization and statistical analysis of results (Mohamad et al., 2020). Du et al. (2020) used factorial design, the speed-riser method, and the response surface method to increase astaxanthin production by 92%.

**FIGURE 2:**
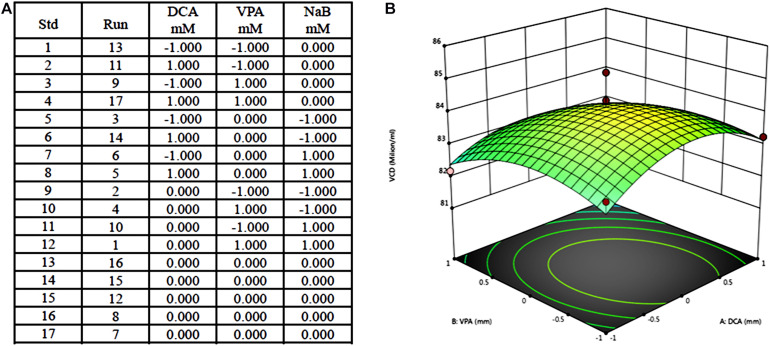
Example of Plackett–Burman design for medium optimization and results. **(A)** Seventeen tests of Plackett–Burman design including three components at two concentration; **(B)** Response surface results of 17 tests.

An example for the design and results of PBM includes three components, dichloroacetate (DCA), valproic acid (VPA), and NaB, at two concentrations. The three influencing factors in the experiment, DCA, VPA, and NaB, were selected as high and low concentrations, respectively (1 is a high concentration, −1 is a low concentration, and the system automatically gives the intermediate concentration as 0), and 17 kinds of combination test concentrations (Figure 2A) were obtained. According to the obtained combination, a pre-experiment was carried out to obtain the corresponding value of cell density, and the curved response graph was obtained after the mean difference standard deviation calculation (Figure 2B).

## Special Additives and Functions

In recent years, some special alternative additives have been used to increase recombinant protein yields in CHO cells. The more common special additives mainly originate from some small molecule chemical compounds and some effective components of hydrolysate (Kumar et al., 2007). The function and possible mechanism of these additives are as follows (Table 3).

**TABLE 3:** Summary of additives of different mechanism components.

**Mechanism**	**Additive agent**	**MVCC (10^6^ × cells/mL)**	**qmAb (pg/cell/day)**	**MMC (mg/L)**	**References**
Cell growth	DS (15,000 Da)	5.23 ± 0.10	13.7 ± 0.80	390.1 ± 17.5	Park et al., 2016a
	EGCG 7/8 mM	16 ± 0.10	16 ± 0.50	600∼800	Yamano and Omasa, 2018
	IGF-1		>Control		Xie et al., 2018
	Resveratrol 25/50 μM	10 ± 1.20	4.7 ± 0.6	0.203 ± 0.004	Toronjo-Urquiza et al., 2020
	…	…	…	…	…
Cell cycle	valeric acid1.5 mM	1.87 ± 0.05	45.6 ± 5.1	0.87 ± 0.31	Park et al., 2016b
	Catechins	16 ± 0.10	16 ± 0.50	600∼800	Yamano and Omasa, 2018
	VPA 25 mM	∼1 ± 0.55	∼10 ± 1.20	∼200	Backliwal et al., 2008
	…	…	…	…	….
Cell metabolim	Glutamine	2000 ± 0.55	2440 ± 86	87.4 ± 1.2	Pan et al., 2017
	α-KG 2 mM	1.43	6.2 μg	0.0165	Nilsang et al., 2008
	DCA 5 mM	7.47	8.80 ± 0.12	0.138	Buchsteiner et al., 2018
	…	…	…	…	…
Cell apoptosis/autophagy	NaB 5 mM	∼6.15		0.11	Bonnotte et al., 1998; Lee and Lee, 2012
	Suramin 5 mM	5.16 ± 0.1		0.16	Lim et al., 2019
	3-MA 5 mM	three-fold	2.8-fold	2.8-fold	Ghaffari et al., 2014
	…	…	…	…	……
Glycosylation	Balcalein (DMSO) 100 μM	2.35 ± 0.06	22.13 ± 2.20	0.901 ± 0.112	Ha et al., 2020
	Raffinose 30 mM	9.2	1.2 ± 0.22	4.2	Zhang and Knapp, 2018
	Mannose	35	2.13 ± 0.05	9	Slade et al., 2016
	Zinc150 μM	∼5 ± 0.10			Prabhu et al., 2018
	…	…	…	…	….
Multiple mechanism combination	1,3,4-O-Bu_3__–_ManNAc + glycoengineering	∼3	∼1.8-fold	∼2-fold	Yin et al., 2018
	All trans carboxylic acid (100 nM) + NaB (0.5 mM) (30°C)	∼4.6	55 ± 0.03	0.0075 ± 0.0003	Rahimi-Zarchi et al., 2018
	NaB (30°C)	∼1.8	23 ± 0.11	170 mg/L	Chen et al., 2011
	NaB (30°C) + GF	7.9	18.5 ± 4.7	0.3 ± 0.05	Hong et al., 2011
	…	…	…	…	…

*MVCC, Maximum viable cell concentration; MMC, Maximum mAb concentration; α-KG, α-ketoglutarate; 3-MA, 3-methyl adenine; EGCG, epigallocatechin-3-gallate; IGF-1, Insulin-like GF-I.*

### Cell Growth Regulation

Some additives can regulate CHO cell growth during recombinant protein production. Dextran sulfate (DS) can increase the nucleic acid hybridization rate and inhibit DNA polymerase activity. The addition of DS to CHO cell cultures, regardless of the molecular weights and concentration of DS used, resulted in the improvement of cell growth and viability. The effect of the addition of DS on the mass properties of the mAbs depends on the molecular weight and concentration of the DS, cell lines, and/or mAbs expressed. However, the addition of DS had no negative effects on three mass properties of the two cell lines; aggregation, charge change, and N-glycosylation of mAbs. Regardless of the molecular weight and concentration used, the addition of DS did not affect the N-glycosylation of the mAbs of the two cell lines (Park et al., 2016a). The addition of epigallocatechin-3-gallate cell culture medium resulted in slower cell growth, longer culture time, and improved specific productivity and total yield of the recombinant IgG1 in batch culture by nearly 50% for an additional 2 or 3 days of culture. There were no other glycoform changes upon 8 μM epigallocatechin-3-gallate addition, which had no influence on the antibody quality (Yamano and Omasa, 2018; Kaneyoshi et al., 2019b). Insulin-like GF-I can replace insulin, and its activity is close to its physiological concentration, suggesting that it may replace GF, regulate cell growth, and promote cell proliferation (Adamson and Walum, 2007; Xie et al., 2018). In total, 25–50 mM resveratrol can slow cell growth and nearly double cell-specific productivity, resulting in a 1.37-fold increase in final IgG titer. Resveratrol acts on CHO cells by slowing the cell growth and increasing the cell-specific protein productivity without changing the characteristics of the IgG produced (Toronjo-Urquiza et al., 2020).

### Cell Cycle Regulation

In the cell cycle, the progression from the G1 phase to S phase and G2 phase to M phase are two points of the cell cycle. Some common cyclin-regulating proteins are found in tumor cells, such as p27 and cyclin D1, which are cyclin-dependent kinase inhibitors. Since p21 and p27 are cyclin-dependent kinase inhibitors, they bind to cyclin-dependent kinase complexes and reduce kinase activity and mediate accumulation of cells in the G0-G1 phase (Yagi et al., 2010; Zhang et al., 2019). P21 inhibitors (CDKI, an important cyclin-dependent kinase inhibitor) are expressed at low levels (Du et al., 2015). The beneficial effect of valeric acid on culture longevity and the production of mAbs outweighs its detrimental effect on specific growth rate (μ) by blocking the cell cycle in the G1 phase, resulting in a 2.9-fold increase in the maximum mAb concentration when 1.5 mM of valeric acid was added to the cultures. Valeric acid did not negatively impact the product quality attributes of the mAb with regards to aggregation, charge variants, and N-glycosylation. Rather, valeric acid improved galactosylation of the mAb (Backliwal et al., 2008; Park et al., 2016b). Catechins induced G0/G1 cell cycle arrest to affect the recombinant protein production; glucosylation of catechin required access to a panel of catechin derivatives protected on all hydroxyl groups, except the one to be glucosylated (Yamano and Omasa, 2018). Meanwhile, VPA inhibited both class I and II histone deacetylases (HDACs), with high potency for class I HDACs, and induced cell cycle arrest at the G1 phase. All iHDACs increased transient antibody yield by at least 1.5-fold in CHO-DG44 (Backliwal et al., 2008, Yagi et al., 2010).

### Cell Metabolism Regulation

At the current stage of CHO cell production, the biosynthesis of recombinant proteins is controlled by some cellular processes that are dependent on lipid metabolism (Wu and Näär, 2019; Budge et al., 2020). The distribution of key media components during glycolysis, the tricarboxylic acid cycle, lactate production, and biosynthetic pathways switch dramatically between exponential growth and stationary (production) phases (Dean and Reddy, 2013). Glutamine is both utilized more efficiently than glucose for anaplerotic replenishment and contributes more significantly to lactate production during the exponential phase (Pan et al., 2017). The use of hybridoma SFM reduces production costs, while the addition of α-ketoglutarate promotes cell growth and mAb production (Nilsang et al., 2008). Buchsteiner et al. (2018) found that in cultures with DCA, lactate production and glucose consumption during exponential growth were reduced by approximately 40% and 35%, respectively. Culture length was extended by over 3 days, and the final antibody titer increased by more than two-fold.

### Cell Apoptosis and Autophagy

Hyperosmolality in recombinant CHO cell cultures induces autophagy and apoptosis. Overexpression of antiapoptotic Bcl-2 proteins in recombinant CHO cells can inhibit apoptotic cell death induced by hyperosmolality, resulting in a substantial increase in recombinant protein production (Han et al., 2011; Templeton et al., 2014). Human and rat colon cancer cells undergo massive apoptosis when they are exposed to soluble Fas ligand in the presence of NaB, an agent that alone induces a low rate of apoptosis only. NaB does not increase Fas receptor cell surface expression and does not modify cell levels of Bcl-2, Bcl-xL, Bcl-xS, and Bax (Bonnotte et al., 1998; Lee and Lee, 2012). Overall, treatment with 5 mM of NaB increases the cleaved forms of the PARP-, caspase- 3-, and Annexin V-positive populations. Moreover, gambogic acid induces ROS and elicits a strong autophagic response at early time points. Gambogic acid induces autophagy and acts as a cell survival response as well as delays caspase activation (Ishaq et al., 2014). ROS-mediated caspase activation degrades autophagic machinery and induces apoptosis. Suramin can inhibit cell apoptosis and positively affect the yield and quality of fusion protein and has no effect on the aggregation of fusion protein, and the sialic acid content increases by 1.18 times (Lim et al., 2019). The increase in sialization is not due to the increase in nucleotide sugar content but to the inhibition of sialase activity. Through optimizing the timing and dose of 3-methyl adenine treatment, the cell-specific productivity increases four-fold, resulting in a 2.8-fold increase in total mAb production (Ghaffari et al., 2014). The positive effect of the 3-methyl adenine treatment appeared to be reduced when the amino acid feed concentration was increased five-fold. Further investigation revealed that both the cell-specific productivity and the total mAb almost doubled by slowly increasing osmolality up to 450 mOsm/kg.

### Glycosylation

CHO cells are unable to express α3gal epitopes. The α2, 3-sialic acid transferase (3-STS) expression of RTPs produced in CHO cells is achieved by adding α2, 3-linking terminal sialic acid (NEUGC/NEUAC) to N-glycan. However, during cell culture, this enzyme often fails to completely cover all N-glycan branches, leaving a variable amount of free β-galactose residues, which is not conducive to prolonging the cyclic half-life. Therefore, various genetic engineering methods have been developed to enhance the sialylation of glycoproteins in rodent cells; (1) increasing the intracellular sialic acid pool; (2) increasing the availability of the nucleotide precursor CMP-NEUAC; (3) increasing the number of STS receptor sites; (4) overexpressing STS; (5) and reducing the activity of soluble sialidase. These methods can significantly increase the content of sialic acid and salivary endogenous components in cells but have limited effect on therapeutic proteins (Fukuta et al., 2000). The CHO cells also lack the ability to express the α2, 6-STS that transfers sialic acid to the N-linking glycan of the protein at site 6. Since the expression systems of yeasts, plants, insects, or birds cannot carry out the sialylation reaction of 6-chains, 6-chains are the preferred terminal glycosylation site of human proteins, especially those in the blood; thus, they are generally considered typical human glycosylation. Therefore, the α2, *6-STS* activity is necessary for the production of glycoproteins with a human glycoprotein pattern (Ferrari et al., 1998; El Maï et al., 2013).

Glycosylation is a key quality attribute of mAbs and other recombinant proteins because it affects the effect and half-life (Ghaderi et al., 2012; Ha et al., 2019). A variety of compounds can regulate the glycosylation profile of recombinant mAbs produced by CHO cells (Ghaderi et al., 2012). In addition, transcriptomics analysis has shown that cottonose supplementation changes the expression levels of many glycosylation-related genes, with galactose transferase being downregulated and salivary transferase upregulated (Prabhu et al., 2018). Mannose can increase the concentration of GDP-Man, resulting in the inhibition of α-mannosidase. The abnormally high concentration of mannose and its metabolites inhibit α-mannosidase activity, and this inhibition in the ER and Golgi may cause the production of IgG with increased high-mannose glycosylation (Slade et al., 2016; Brühlmann et al., 2017). Cu, as a competitive sialidase inhibitor, can avoid the undesired amide O-glycosylation (Ghaffari et al., 2014; Ha et al., 2020). Zn can increase the galactosylation level and regulate the concentration of trace elements (Zhang and Knapp, 2018).

### Multiple Mechanisms

At present, many studies have used different components to complement and promote each other to work together to achieve the goal of promoting cell growth and improving protein expression quantity and quality (Yang et al., 2017). Compound combinations of additives are also efficient at achieving a smaller overall glycosylation modulation (Reinhart et al., 2015; Wang et al., 2019). In total, 300 μM of dexamethasone and the some mixture of Mn, uridine, and galactose can increase galactose 1,3,4-o-bu_3_-mannac, and combined with sugar engineered GnTIV/GnTV/ST6, salinization is increased by 6.9% (Yin et al., 2018). All-trans carboxylic acid combined with NaB can reduce Rb expression and enhance Rb phosphorylation by downregulating CKIs (Chen et al., 2011; Rahimi-Zarchi et al., 2018). There were no apparent changes regarding the total sialic acid content of the antibody, but manipulation of cultures with NaB treatment and low culture temperature decreased N-glycolylneuraminic acid levels by 5∼10%. The simultaneous application of NaB and a low culture temperature is an effective way to extend the culture period and enhance the final antibody concentration without compromising the sialic acid content or biological activity (Choi et al., 2005; Chen et al., 2011). Meanwhile, Hong et al. (2011) observed that with 3 mM of NaB, cell viability fell below 80% after day four in GF-containing medium, but it remained over 80% until day 18 in GF-deficient medium. Due to the enhanced production of mAb and the extended culture longevity, an approximately two-fold increase in total antibody production was achieved in pseudo-perfusion culture with 1 mM of NaB in GF-deficient medium; the withdrawal of GF in combination with the addition of NaB can be considered a relevant strategy for alleviating NaB-induced cell apoptosis and enhancing antibody production since it can be easily implemented and because it enhances the production of mAb and extend culture longevity.

### Protein Purification

There are two common methods for protein purification; (1) Ni affinity chromatography, wherein Ni is eluted by the binding of Ni ion and 6 × His tag protein; and (2) ion exchange chromatography, usually for the purification of biological macromolecules under certain pH conditions and the separation of proteins with different charges. The mAbs are purified by combining the Fc fusion fragment with Protein A. In the CHO SFM, metals are trace element ions that do not include Ni ions; thus, downstream purification, such as 6 × His tag protein, is not affected in the later stage. At the same time, because CHO SFM has a stable osmotic pressure and certain pH range, it does not have high impact on ion exchange. In general, the related components and parameters of CHO SFM have no significant effect on protein purification.

## Conclusion and Future Perspectives

With increasing demand for recombinant protein products, an increasing number of good quality SFM types for CHO cells are needed. At present, five types of SFM are available, and CDM is the most ideal medium due to its chemically defined components. The key components of SFM include energy sources, amino acids, vitamins, lipids, trace elements, inorganic salts, anti-shear protectants, and nucleic acids. Different media contain different formulations and have been optimized; thus, they can adapt to the growth of different cell lines and the production of recombinant protein. Based on the characteristics of CHO cells and its advantages in RTP production, the key components of SFM have been optimized to different degrees and proportions, and experimental designs have been prepared. PBM and the response surface method are commonly used for SFM optimization, which can be combined with other statistical methods, such as non-parametric Gaussian kernel regression and partial least squares regression, to optimize the SFM for a higher titer of recombinant mAbs.

In recent years, studies have found that some small molecule compound additives can improve the yield and quality of RTPs. The function and possible mechanisms may involve cell growth regulation, cell metabolism regulation, cell apoptosis and autophagy, glycosylation, and multiple mechanisms.

Recently, considerable progress has been made in CHO cell SFM, and various types of commercial SFM are available. However, most SFM for CHO cells contain yeast or plant hydrolysates; unlike CDM, the components are not clear and defined. In addition, viable cell density and recombinant protein production must be increased to meet the demand for recombinant proteins and reduced cost. With increasing understanding and the use of new cell biotechnologies such as modern multi-omics and the emergence of new process monitoring and analysis technologies, optimal design of SFM for CHO cells will result in considerable and widespread progress in a variety of fields.

## Author Contributions

WL: manuscript preparation and revision. ZF: proofread and manuscript revision. YL: manuscript revision. T-YW: manuscript design and revision.

## Conflict of Interest

The authors declare that the research was conducted in the absence of any commercial or financial relationships that could be construed as a potential conflict of interest.
